# Tamoxifen Use Correlates with Increased Risk of the First Episode of Ischemic Cerebrovascular Disease in Older Women with Breast Cancer: A Case-Control Study in Taiwan

**DOI:** 10.3389/fphar.2017.00742

**Published:** 2017-10-17

**Authors:** Shih-Wei Lai, Cheng-Li Lin, Kuan-Fu Liao

**Affiliations:** ^1^Department of Medicine, China Medical University, Taichung, Taiwan; ^2^Department of Family Medicine, China Medical University Hospital, Taichung, Taiwan; ^3^Management Office for Health Data, China Medical University Hospital, Taichung, Taiwan; ^4^Department of Medicine, Tzu Chi University, Hualien, Taiwan; ^5^Department of Internal Medicine, Taichung Tzu Chi General Hospital, Taichung, Taiwan

**Keywords:** breast cancer, ischemic cerebrovascular disease, woman, Taiwan National Health Insurance Program, tamoxifen

## Abstract

**Background and Objectives:** There are inconsistent results about the association between ischemic cerebrovascular disease and tamoxifen use in women with breast cancer. The study aimed to evaluate the association between the risk of ischemic cerebrovascular disease and tamoxifen use in older women with breast cancer in Taiwan.

**Methods:** We designed a retrospective, nationwide, case-control study using the database of the Taiwan National Health Insurance Program. A total of 800 female subjects with breast cancer aged ≥65 years with the first episode of ischemic cerebrovascular disease from 2000 to 2011 were identified as the cases. Additionally, 2,876 female subjects with breast cancer aged ≥65 years without any type of cerebrovascular diseases were selected as the control subjects. The cases and the control subjects were matched with age and comorbidities. Ever use of tamoxifen was defined as a subject who had at least a prescription for tamoxifen before the index date. Never use of tamoxifen was defined as a subject who never had a prescription for tamoxifen before the index date. We used the multivariable logistic regression model to calculate the odds ratio (OR) and 95% confidence interval (CI) for ischemic cerebrovascular disease associated with tamoxifen use.

**Results:** After adjusting for confounding variables, the adjusted OR of ischemic cerebrovascular disease was 2.5 for subjects with ever use of tamoxifen (95% CI 2.10, 2.97), compared with never use of tamoxifen. In addition, the adjusted OR of ischemic cerebrovascular disease was 1.15 (95% CI 1.10, 1.21) in subjects with ever use of tamoxifen as increase in use duration per 1 year. The adjusted OR of ischemic cerebrovascular disease was 2.54 (95% CI 2.03, 3.17) in subjects with ever use of tamoxifen as increase in dosage per 1 mg.

**Conclusions:** Tamoxifen use is significantly associated with 2.5-fold increased odds of ischemic cerebrovascular disease among older women with breast cancer in Taiwan. There are duration-dependent and dose-dependent effects of tamoxifen use on the risk of ischemic cerebrovascular disease.

## Introduction

Tamoxifen is commonly used for prevention of breast cancer in healthy women at elevated risk and it has good preventive effects (Cuzick et al., 2013, 2015). Tamoxifen use is associated with increased risk of venous thromboembolism in women with breast cancer, (Deitcher and Gomes, 2004) but the influence of tamoxifen use on the risk of ischemic cerebrovascular disease remains unsettled. Some animal studies showed that tamoxifen use has a neuroprotective effect in cerebral ischemia (Kimelberg et al., 2000; Mehta et al., 2003; Zhang et al., 2007; Wakade et al., 2008; Boulos et al., 2011). Epidemiological studies showed inconsistent results about tamoxifen use on the risk of ischemic cerebrovascular disease, including reduced risk, (Yang et al., 2014) increased risk, (Bushnell and Goldstein, 2004; Hooning et al., 2006) and no association (Geiger et al., 2004).

Cerebrovascular disease was the fourth leading cause of total deaths in women in Taiwan in 2016 (4,930 deaths due to cerebrovascular disease, 7.1% of 69,433 total deaths in women) (Taiwan Ministry of Health and Welfare, 2016a). Breast cancer was the fourth leading cause of cancer deaths in women in Taiwan in 2016 (2,176 deaths due to breast cancer, 11.7% of 18,545 total cancer deaths in women) (Taiwan Ministry of Health and Welfare, 2016a). Little evidence is available about the effect of tamoxifen use on the risk of ischemic cerebrovascular disease in Taiwan. Therefore, we designed a retrospective, nationwide, case-control study to evaluate the association between the risk of ischemic cerebrovascular disease and tamoxifen use in older women with breast cancer in Taiwan. Due to the incidence of the outcome (ischemic cerebrovascular disease) being probably low, that is why a case-control study, rather than a cohort study, was designed.

## Methods

### Study design and data source

We designed a retrospective, nationwide, case-control study to analyze the database of the Taiwan National Health Insurance Program. Taiwan is an independent country with more than 23 million people (Chao et al., 2015; Chen et al., 2015; Ho and Chang, 2015; Hsiao et al., 2015; Hung and Ku, 2015; Jao et al., 2015; Chen and Wu, 2016; Chen S. Y. et al., 2016; Chen Y. F. et al., 2016; Liang et al., 2017; Liao et al., 2017a; Wen and Yin, 2017). This program started in March 1995 and has covered 99.6% of the entire population of 23 million people living in Taiwan by the end of 2015 (Taiwan Ministry of Health Welfare, 2016b). The details of the program can be found in previous studies (Lai et al., 2010, 2013; Liao et al., 2012; Yang et al., 2015; Chen H. Y. et al., 2016; Chu et al., 2017). The study was approved by the Research Ethics Committee of China Medical University and Hospital in Taiwan (CMUH-104-REC2-115).

### Sampled subjects

Female subjects with breast cancer aged ≥65 years with the first episode of ischemic cerebrovascular disease (ICD-9 codes 433, 434, and 435) from 2000 to 2011 were identified as the cases. The date of a subject being diagnosed with the first episode of ischemic cerebrovascular disease was defined as the index date. Additionally, for every one case with ischemic cerebrovascular disease, approximately three female subjects with breast cancer aged ≥65 years who had never been diagnosed with any type of cerebrovascular diseases were randomly selected from the same database as the control subjects. The cases and the control subjects were matched with age (5-year interval), comorbidities, and the year of index date.

### Comorbidities

Comorbidities which could be potentially related to ischemic cerebrovascular disease before the index date were identified as follows: alcohol-related disease, atrial fibrillation, chronic kidney disease, chronic obstructive pulmonary disease, coronary artery disease, diabetes mellitus, hyperlipidemia, and hypertension. Based on the ICD-9 codes, the diagnosis accuracy of comorbidities was well evaluated in previous studies (Wong et al., 2016; Lai et al., 2017b,a,d; Lin et al., 2017a,b).

### Measurements of tamoxifen use and aromatase inhibitors use

Prescription histories of tamoxifen and aromatase inhibitors were included in the study. The definition of medication use was adapted from previous studies (Cheng et al., 2017; Lai et al., 2017e,c; Liao et al., 2017b,c). Ever use of medications was defined as a subject who had at least a prescription for medications studied before the index date. Never use of medications was defined as a subject who never had a prescription for medications studied before the index date.

### Statistical analysis

We compared the distributions of the demographic status, tamoxifen use, aromatase inhibitors use, and comorbidities between the cases and the control subjects using the χ^2^ test for categorized variables and the *t*-test for continuous variables. Variables which were significantly associated with ischemic cerebrovascular disease in the univariable logistic regression model were further examined by the multivariable logistic regression model. The odds ratio (OR) and 95% confidence interval (CI) were used to estimate the risk of ischemic cerebrovascular disease associated with tamoxifen use. The risk of ischemic cerebrovascular disease associated with cumulative duration and cumulative dosage of tamoxifen use were also estimated. All analyses were performed using SAS statistical software (version 9.2; SAS Institute, Inc., Cary, NC, USA). The results were considered statistically significant when two-tailed *P*-values were <0.05.

## Results

### Characteristics of the study population

Table 1 demonstrates the characteristics of the study population. We identified 800 cases with the first episode of ischemic cerebrovascular disease in 2000–2011 and 2,876 control subjects. The mean ages (standard deviation) were 78.6 (6.38) years in cases and 77.8 (6.22) years in control subjects, with statistical significance (*t*-test, *P* = 0.001). The mean durations of tamoxifen use (standard deviation) was 2.03 (1.81) years in cases and 1.98 (1.79) years in control subjects, without statistical significance (*t*-test, *P* = 0.57). The cases with ischemic cerebrovascular disease were more likely to have a higher proportion of ever use of tamoxifen than the control subjects (71.0 vs. 49.4%, χ^2^ test, *P* < 0.001). The cases had significantly higher proportions of alcohol-related disease and atrial fibrillation than the control subjects (χ^2^ test, 1.75% vs. 0.52%, *P* = 0.001, and 14% vs. 7.71%, *P* = 0.001, respectively). There was no significant difference in the distributions of ever use of aromatase inhibitors and other comorbidities between the cases and the control subjects (χ^2^ test, *P* > 0.05 for all).

**Table 1:** Characteristics between cases with ischemic cerebrovascular disease and control subjects.

	**Control subjects *N* = 2876**		**Cases with ischemic cerebrovascular disease *N* = 800**		
**Variable**	***n***	**(%)**	***n***	**(%)**	*P*-value^*^
Age group (years)					0.40
65–74	919	32.0	243	30.4	
75–84	1,534	53.3	425	53.1	
≥85	423	14.7	132	16.5	
Age (years), mean (standard deviation) ^†^	77.8	6.22	78.6	6.38	0.001
Duration of exposure to tamoxifen (years), mean (standard deviation)^†^	1.98	1.79	2.03	1.81	0.57
Ever use of tamoxifen	1420	49.4	568	71.0	<0.001
Ever use of aromatase inhibitors	430	15.0	139	17.4	0.09
COMORBIDITIES^*^					
Alcohol-related disease	15	0.52	14	1.75	0.001
Atrial fibrillation	222	7.71	112	14.0	0.001
Chronic kidney disease	139	4.83	51	6.38	0.08
Chronic obstructive pulmonary disease	696	24.2	205	25.6	0.41
Coronary artery disease	1,536	53.4	438	54.8	0.5
Diabetes mellitus	1,185	41.2	330	41.3	0.98
Hyperlipidemia	1,368	47.6	380	47.5	0.97
Hypertension	2,611	90.8	720	90.0	0.5

*Data are presented as the number of subjects in each group with percentages given in parentheses, or mean with standard deviation given in parentheses*.

* χ^2^ test and

† *t-test comparing subjects with and without ischemic cerebrovascular disease*.

### Risk of ischemic cerebrovascular disease associated with tamoxifen use, aromatase inhibitors use, and comorbidities

Table 2 demonstrates the risk of ischemic cerebrovascular disease associated with tamoxifen use, aromatase inhibitors use, and comorbidities. After adjusting for confounding variables, the multivariable logistic regression model demonstrated that the adjusted OR of ischemic cerebrovascular disease was 2.5 for subjects with ever use of tamoxifen (95% CI 2.10, 2.97), compared with never use of tamoxifen. In addition, alcohol-related disease (adjusted OR 4.16, 95% CI 1.96, 8.87), and atrial fibrillation (adjusted OR 2.0, 95% CI 1.56, 2.56) were also associated with ischemic cerebrovascular disease.

**Table 2:** Crude and adjusted odds ratio and 95% confidence interval of ischemic cerebrovascular disease associated with tamoxifen use, aromatase inhibitors use, and comorbidities.

**Variable**	**Crude**		**Adjusted^†^**	
	**OR**	**95%CI**	**OR**	**95%CI**
Age (per one year)	1.02	1.01, 1.03	1.01	0.99, 1.02
Tamoxifen (never use as a reference)				
Ever use	2.51	2.12, 2.97	2.5	2.10, 2.97
Aromatase inhibitors (never use as a reference)				
Ever use	1.20	0.97, 1.48		
**COMORBIDITIES (YES vs. NO)**				
Alcohol-related disease	3.40	1.63, 7.07	4.16	1.96, 8.87
Atrial fibrillation	1.95	1.53, 2.48	2.0	1.56, 2.56
Chronic kidney disease	1.34	0.96, 1.87		
Chronic obstructive pulmonary disease	1.08	0.90, 1.29		
Coronary artery disease	1.06	0.90, 1.24		
Diabetes mellitus	1.00	0.86, 1.18		
Hyperlipidemia	1.00	0.85, 1.17		
Hypertension	0.91	0.70, 1.19		

† *Variables found to be statistically significant in the univariable logistic regression model were further examined by the multivariable logistic regression model. Adjusting for age, alcohol-related disease, and atrial fibrillation*.

Because subjects with alcohol-related disease had the highest odds of ischemic cerebrovascular disease, we made a sub-analysis of interaction effects on the risk of ischemic cerebrovascular disease between tamoxifen use and alcohol-related disease. When compared with subjects with never use of tamoxifen and without alcohol-related disease, the adjusted OR of ischemic cerebrovascular disease was 2.51 (95% CI 2.11, 2.99) among subjects with ever use of tamoxifen and without alcohol-related disease. The adjusted OR increased to 7.94 (95% CI 2.63, 24.0) among subjects with ever use of tamoxifen and with alcohol-related disease.

### Risk of ischemic cerebrovascular disease associated with cumulative duration of tamoxifen use

Table 3 demonstrates the effect of cumulative duration of tamoxifen use on the risk of ischemic cerebrovascular disease in multivariable logistical regression model. Compared with never use of tamoxifen, the adjusted OR of ischemic cerebrovascular disease was 1.15 (95% CI 1.10, 1.21) in subjects with ever use of tamoxifen as increase in use duration per 1 year. The sub-analysis demonstrated that the adjusted ORs of ischemic cerebrovascular disease were 2.45 (95% CI 2.04, 2.95) for subjects with cumulative duration of tamoxifen use <3 years, and 2.61 (95% CI 2.07, 3.29) for subjects with cumulative duration of tamoxifen use ≥3 years, compared with never use of tamoxifen.

**Table 3:** The risk of ischemic cerebrovascular disease associated with cumulative duration of tamoxifen use.

**Variable**	**Case number /control number**	**Crude OR**	**95% CI**	**Adjusted OR^†^**	**95% CI**
Never use of tamoxifen as a reference	232/1456	1.00	reference	1.00	reference
Cumulative duration of tamoxifen use (increase in duration per 1 year)	568/1420	1.17	1.12, 1.22	1.15	1.10, 1.21

† *Variables found to be statistically significant in the univariable logistic regression model were further examined by the multivariable logistic regression model. Adjusting for age, alcohol-related disease, and atrial fibrillation*.

### Risk of ischemic cerebrovascular disease associated with cumulative dosage of tamoxifen use

Table 4 demonstrates the effect of cumulative dosage of tamoxifen use on the risk of ischemic cerebrovascular disease in multivariable logistical regression model. Compared with never use of tamoxifen, the adjusted OR of ischemic cerebrovascular disease was 2.54 (95% CI 2.03, 3.17) in subjects with ever use of tamoxifen as increase in dosage per 1 mg. According to the median dose, the sub-analysis demonstrated that the adjusted ORs of ischemic cerebrovascular disease were 2.37 for subjects with average daily dose of tamoxifen use <20 mg (95% CI 1.98, 2.85), and 2.92 for subjects with average daily dose of tamoxifen use ≥20 mg (95% CI 2.29, 3.72), compared with never use of tamoxifen.

**Table 4:** The risk of ischemic cerebrovascular disease associated with cumulative dosage of tamoxifen use.

**Variable**	**Case number /control number**	**Crude OR**	**95% CI**	**Adjusted OR^†^**	**95% CI**
Never use of tamoxifen as a reference	232/1456	1.00	reference	1.00	reference
Cumulative dosage of tamoxifen use (increase in dosage per 1 mg)	568/1420	2.57	2.07, 3.21	2.54	2.03, 3.17

† *Variables found to be statistically significant in the univariable logistic regression model were further examined by the multivariable logistic regression model. Adjusting for age, alcohol-related disease, and atrial fibrillation*.

## Discussion

In this retrospective case-control study, we noticed that tamoxifen use was significantly associated with increased odds of ischemic cerebrovascular disease (Table 2). We noticed that there seems to be a duration-dependent effect of tamoxifen use on the risk of ischemic cerebrovascular disease (Table 3). If the cumulative duration of tamoxifen use was 3 years or longer, the odds would be increased. That is, the longer the tamoxifen use, the greater the risk of ischemic cerebrovascular disease. We noticed that there seems to be a dose-dependent effect of tamoxifen use on the risk of ischemic cerebrovascular disease (Table 4). If the average daily dose of tamoxifen use was 20 mg or more, the odds would be increased. That is, the higher the average daily dose of tamoxifen use, the greater the risk of ischemic cerebrovascular disease. Our findings are compatible with previous studies showing that tamoxifen use was significantly associated with increased odds of ischemic cerebrovascular disease (adjusted OR 1.82–1.88), (Bushnell and Goldstein, 2004; Hooning et al., 2006) but contrary to Yang et al's study showing that tamoxifen use was significantly associated with reduced hazard of ischemic cerebrovascular disease (adjusted HR 0.52, 95% CI 0.35, 0.78) (Yang et al., 2014). In Yang et al's study, the index date seems to be the date of a subject being diagnosed with breast cancer, rather than the date of tamoxifen being prescribed. Therefore, immortal time bias substantially exists. That is, the reduced hazard might be confounded by immortal time bias. Our study is a case-control study. Thus, immortal time bias can be minimized.

We noticed that subjects with ever use of tamoxifen and without alcohol-related disease were associated with 2.51-fold increased odds of ischemic cerebrovascular disease, compared with subjects with never use of tamoxifen and without alcohol-related disease. This finding suggests that the risk of ischemic cerebrovascular disease associated with tamoxifen use is independent of alcohol-related disease. Tamoxifen use has a pivotal role on the risk of ischemic cerebrovascular disease. The adjusted OR increased to 7.94 among subjects with ever use of tamoxifen and with alcohol-related disease. This finding suggests that there is an interaction effect on the risk of ischemic cerebrovascular disease between tamoxifen use and alcohol-related disease.

## Limitation

Some limitations should be discussed. First, a causal-relationship cannot be established in a case-control study. Second, due to only observational studies available, the underlying biological mechanism of the association between ischemic cerebrovascular disease and tamoxifen use cannot be fully elucidated. Currently, there is not definite evidence to elucidate the mechanism, but estrogen-like prothrombotic effect of tamoxifen may be partly responsible for the positive association between ischemic cerebrovascular disease and tamoxifen use (Bushnell and Goldstein, 2004; Decensi et al., 2005). That is, the estrogen-like prothrombotic effect of tamoxifen potentially causes venous thrombosis, such as cerebral sinus thrombosis, deep vein thrombosis, or pulmonary embolism, which further develops paradoxical embolism (Akdal et al., 2001; Cramer et al., 2004; Masjuan et al., 2004; Ueno et al., 2007). Paradoxical embolism enters arterial system via patent foramen ovule and then flows to intracranial circulation (Bogousslavsky et al., 1996; Lapostolle et al., 2003; Desai et al., 2006; Tanislav et al., 2011). Thus, ischemic cerebrovascular disease occurs. Due to having conflicting results in previous studies, more prospective cohort studies are required to elucidate this issue.

## Strength

It is an interesting study based on a well-organized health care database in an Asiatic country. The study is well-conducted with taking care of a proper study design and the study has sufficient control subjects. It appears to be informative and influential on the association between ischemic cerebrovascular disease and tamoxifen use in women with breast cancer.

## Conclusion

We conclude that tamoxifen use is significantly associated with 2.5-fold increased odds of ischemic cerebrovascular disease in older women with breast cancer in Taiwan. There are duration-dependent and dose-dependent effects of tamoxifen use on the risk of ischemic cerebrovascular disease.

## Author contributions

SL planned and conducted this study. He contributed to the conception of the article, initiated the draft of the article, and revised the article. CL conducted the data analysis and revised the article. KL planned and conducted this study. He participated in the data and revised the article.

### Conflict of interest statement

The authors declare that the research was conducted in the absence of any commercial or financial relationships that could be construed as a potential conflict of interest.
